# Zusammenhang von körperlicher Aktivität mit Fatigue und Funktionskapazität bei Patienten mit rheumatoider Arthritis

**DOI:** 10.1007/s00393-020-00830-2

**Published:** 2020-06-25

**Authors:** S. Beider, S. Flohr, S. Gehlert, T. Witte, D. Ernst

**Affiliations:** 1 Klinik für Rheumatologie und Immunologie, Medizinische Hochschule Hannover, Carl-Neuberg-Str. 1, 30625 Hannover, Deutschland; 2 Institut für Sportwissenschaft, Universität Hildesheim, Hildesheim, Deutschland

**Keywords:** Sportliche Aktivität, Empfehlungen, Entzündlich-rheumatische Erkrankungen, Bewegungsmotivation, Müdigkeit, Exercises, Recommendations, Rheumatic diseases, Motivation, Multidimensional assessment of fatigue (MAF)

## Abstract

**Hintergrund:**

Patienten mit rheumatoider Arthritis (RA) neigen dazu, körperlich weniger aktiv zu sein. Die körperliche Aktivität wirkt sich positiv auf die Krankheitsaktivität (KA) und Lebensqualität aus und wird von der European League Against Rheumatism (EULAR) als ein Teil der Standardtherapie empfohlen.

**Fragestellung:**

Eine Querschnittanalyse der RA-Patienten wurde in Bezug auf die KA, die Funktionskapazität (FK) und Fatigue im Zusammenhang mit der körperlichen Aktivität durchgeführt.

**Material und Methoden:**

Die körperliche Aktivität, die FK und der globale Fatigue-Index (Global Fatigue Index [GFI]) wurden mittels standardisierter Fragebögen ermittelt: International Physical Activity Questionnaire-short form (IPAQ-SF), Funktionsfragebogen Hannover (FFbH) und Multidimensional Assessment of Fatigue (MAF). Die Daten wurden mittels SPSS Version 26 (IBM, Armonk, NY, USA) ausgewertet. Die Signifikanzprüfung erfolgte mittels bivariater und partieller Korrelation und nichtparametrischer Tests.

**Ergebnisse:**

Insgesamt wurden 164 Patienten in die Untersuchung eingeschlossen. Die Mehrheit der Patienten war weiblich (127/164; 77 %), das Durchschnittsalter der Kohorte betrug 58,3 (21 bis 86) Jahre. Die durchschnittliche Dauer der krankheitsrelevanten Symptome war 169 (0 bis 713) Monate; 39 % der Patienten zeigten eine niedrige, 37 % eine moderate und 24 % eine hohe körperliche Aktivität. Patienten mit hoher körperlicher Aktivität wiesen die niedrigsten Werte des GFI (*p* < 0,001), eine unbeeinträchtigte FK (*p* < 0,001) und die niedrigste KA (*p* = 0,045) auf.

**Schlussfolgerungen:**

Ein signifikanter Zusammenhang der körperlichen Aktivität mit der Funktionskapazität und der Ausprägung der Fatigue bei RA-Patienten wurde nachgewiesen. Um den Anteil der Patienten mit niedriger körperlicher Aktivität zu senken, sollten die Möglichkeiten des Funktionstrainings ausgeschöpft und die Patienten zu sportlichen Aktivitäten motiviert werden.

**Zusatzmaterial online:**

Die Online-Version dieses Artikels (10.1007/s00393-020-00830-2) enthält weitere Erklärungen zur Auswertung der Fragebögen. Beitrag und Zusatzmaterial stehen Ihnen auf www.springermedizin.de zur Verfügung. Bitte geben Sie dort den Beitragstitel in die Suche ein, das Zusatzmaterial finden Sie beim Beitrag unter „Ergänzende Inhalte“.

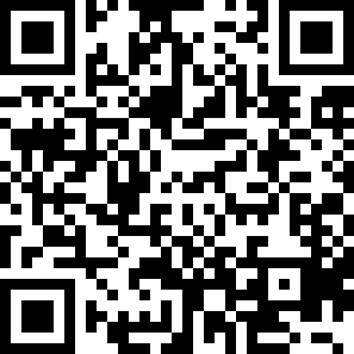

## Abstract (en)

**Background:**

Patients with rheumatoid arthritis (RA) tend to be less physically active. Physical activity has been shown to have a positive impact on disease activity and quality of life and is recommended by the European League Against Rheumatism (EULAR) as an integral component of standard treatment.

**Objective:**

A cross-sectional analysis of RA patients was carried out assessing disease activity, functional capacity and fatigue associated with physical activity.

**Material and methods:**

Physical activity, functional capacity and the global fatigue index (GFI) were examined using standardized questionnaires: the international physical activity questionnaire short form (IPAQ-SF), the functional questionnaire Hannover (FFbH) and the multidimensional assessment of fatigue (MAF). The data were evaluated using SPSS 26 (IBM, Armonk, NY, USA). The level of significance was tested with bivariate and partial correlations and nonparametric tests.

**Results:**

In total 164 patients were included in the study. The majority of the patients were female (127/164; 77%) and the median age of the cohort was 58.3 years (range 21–86 years). The median duration of disease-related symptoms at inclusion was 169 months (range 0–713 months). Physical activity was low in 39%, moderate in 37% and high in 24%. Patients reporting higher levels of physical activity reported significantly lower GFI (*p* < 0.001), functional limitations (*p* < 0.001) and disease activity (*p* = 0.045) scores than those with less physical activity.

**Conclusion:**

Physical activity in RA patients was significantly correlated with functional capacity and levels of fatigue. In order to reduce the proportion of patients with low physical activity, the possibilities for functional training should be expanded and the patients should be encouraged to undertake sporting activities.

Der aktive Lebensstil und die körperliche Aktivität sind als Prädiktoren einer günstigen Prognose bei vielen Erkrankungen anerkannt, darunter auch bei rheumatoider Arthritis (RA). Entsprechend den aktuellen Empfehlungen der European League Against Rheumatism (EULAR) soll die körperliche Aktivität einen integrativen Teil der Standardtherapie darstellen. In der täglichen Praxis ist es wichtig, das Aktivitätsniveau der Patienten zu kennen, die Möglichkeiten der Physiotherapie, des Funktionstrainings und des Rehasports auszuschöpfen und die Patienten zur sportlichen Betätigung zu motivieren.

Die rheumatoide Arthritis (RA) ist die häufigste, chronisch verlaufende, autoimmune Gelenkerkrankung, die mit einer deutlichen Morbidität, sinkender Lebensqualität und steigender Mortalität assoziiert ist [25]. Die Mortalitätserhöhung ist durch Entzündung und Komorbidität, v. a. durch das hohe Risiko der kardiovaskulären Erkrankungen bedingt [1]. Eine führende Ursache von Herz- und Kreislauferkrankungen, auch in der Gesamtpopulation, ist die körperliche Inaktivität. Diese ist außerdem die vierthäufigste Todesursache (6 % aller Todesfälle) weltweit. Es wird dennoch berichtet, dass große Populationsanteile in den Industrieländern ein inaktives Leben führen [18]. RA-Patienten leiden unter Schmerzen und oft eingeschränkter Funktion der betroffenen Gelenke, sie neigen dazu, weniger körperlich aktiv zu sein, als es empfohlen wird, und laufen mit der Zeit Gefahr einer kompletten körperlichen Inaktivität [10, 28]. Die Fatigue stellt ein klinisch relevantes, bei Patienten mit rheumatoider Arthritis sehr häufig vorkommendes (in ca. 40 % der Fälle) Symptom dar [10, 21]. In früheren Studien konnte gezeigt werden, dass sportliche Aktivität einen positiven Nutzen auf die Fatigue haben kann [13]. Sie trägt außerdem zur Prävention vieler onkologischer Erkrankungen, von Schlaganfällen, Diabetes sowie von kardiovaskulären Erkrankungen und Übergewicht bei [9, 20, 26]. Die EULAR-Empfehlungen sehen eine tägliche moderate körperliche Aktivität von mindestens 30 min vor [21].

Ziel dieser Querschnittanalyse war es, die sportliche Aktivität, Fatigue und Krankheitsaktivität (KA) von Patienten mit rheumatoider Arthritis zu erfassen, um herauszufinden, ob sportliche Aktivität mit der KA oder Fatigue korreliert und ob die aktuellen Empfehlungen schon umgesetzt werden.

## Methoden

Patienten mit rheumatoider Arthritis, die vom 01.06.2018 bis zum 31.03.2019 im Rahmen ihrer regelmäßigen Kontrolluntersuchungen die rheumatologische Ambulanz der Medizinischen Hochschule Hannover (MHH) aufgesucht haben, wurden bei der Anmeldung gefragt, ob sie an der Querschnittstudie teilnehmen möchten. Die Teilnahme war freiwillig. Die Patienten wurden konsekutiv erfasst. Alle Teilnehmer wurden über die Studie schriftlich und mündlich aufgeklärt und haben ihr schriftliches Einverständnis gegeben. Ein Ethikvotum mit der Nummer 8179_BO_S_2018 liegt vor. Im Rahmen des Projekts wurden alle teilnehmenden Patienten zu ihrer körperlichen Aktivität befragt. Neben der routinemäßigen Datenerhebung wurden die Patienten mittels definierter Fragebögen zu ihrer Funktionskapazität, der Fatigue und zum Umfang ihrer körperlichen Aktivität in den vergangenen 7 Tagen befragt.

Bei der Datenerhebung wurden anthropometrische Parameter wie Alter, Geschlecht, Gewicht, Größe und Body-Mass-Index (BMI) berücksichtigt. Erhoben wurden außerdem Angaben zur Ernährung, zum Alkoholkonsum und Rauchen, zu sportlichen Aktivitäten in der Vergangenheit und aktuell. Die Krankheitsaktivität wurde mittels Disease Activity Score 28 (DAS28) bewertet. Der Grad der aktuellen körperlichen Aktivität wurde mithilfe des International Physical Activity Score-short form (IPAQ-SF) evaluiert. Dabei wurde zunächst die wöchentliche Metabolic Equivalent Task(MET, 1 MET = 1 Kilokalorie pro Kilogramm Körpergewicht in 1 h [1 kcal/kg/h])-Minuten-Zahl ausgerechnet und anhand definierter Kriterien je nach Art, Dauer und Häufigkeit der Aktivitäten der Aktivitätsgrad bestimmt (s. Tab. 1).

**Table 1:** 

Aktivität	Kriterien
Hohe	Eines der folgenden 2 Kriterien: 1. Anstrengende Aktivitäten an mindestens 3 Tagen *und* MET-Minuten/Woche ergeben zusammengerechnet mindestens 1500 *oder* 2. Alle angegebenen Tage jeglicher Aktivitäten (Gehen, moderat, anstrengend) ergeben aufsummiert 7 oder mehr Tage *und* mindestens 3000 MET-Minuten/Woche
Moderate	Eines der folgenden 3 Kriterien: 1. 3 oder mehr Tage, an denen mindestens 20 min lang anstrengende Aktivitäten durchgeführt wurden *oder* 2. 5 oder mehr Tage, an denen mindestens 30 min pro Tag moderate und/oder gehende Aktivitäten durchgeführt wurden, *oder* 3. 5 oder mehr Tage an denen jegliche Aktivitäten (Gehen, moderat, anstrengend) zusammen mindestens 600 MET-Minuten/Woche ergeben
Niedrige	Eines der folgenden 2 Kriterien: 1. Es wurde gar keine Aktivität angegeben, *oder* 2. Es wurden Aktivitäten angegeben, allerdings nicht genug, um den jeweiligen Kriterien der Kategorien MODERATE oder HOHE zu entsprechen

*MET* Metabolic Equivalent Task

Die Ausprägung der Fatigue wurde mittels Multidimensional Assessment of Fatigue (MAF) erhoben (s. Zusatzmaterial online). Dabei wurde der globale Fatigue-Index (GFI) errechnet. Die Kalkulation ergibt einen Punktwert im Bereich von 1 (keine Müdigkeit) bis 50 (maximale Müdigkeit). In der Literatur finden sich Hinweise, dass der durchschnittliche GFI-Wert bei Patienten mit rheumatoider Arthritis zwischen 26 und 29 liegt, während er bei gesunden Kontrollpersonen zwischen 16 und 17 ist [4]. Für die vorliegende Arbeit wurden Werte des GFI <20 als unauffällig definiert.

Zur Bestimmung der Funktionskapazität wurde der Funktionsfragebogen Hannover (FFbH) angewandt (s. Zusatzmaterial online). Im Ergebnis wird die Funktionskapazität als Gesamtwert von 0 % (minimale Funktionskapazität) bis 100 % (maximale Funktionskapazität) ausgegeben [5]. Eine klinisch relevante Funktionsbeeinträchtigung ist bei Funktionskapazitätswerten unter 60 % anzunehmen, während Werte zwischen 60 und 70 % als auffälliger Befund und Werte zwischen 70 und 80 % als mäßige Funktionsbeeinträchtigung gedeutet werden. Werte über 80 % werden als normale Funktionskapazität verstanden [17, 22].

Die erhobenen Daten wurden zunächst in einer Access-Datenbank gesammelt und durch gezielte Abfragen in eine SPSS 26-Datenbank (IBM, Armonk, NY, USA) überführt. Die Auswertung beinhaltete deskriptive Statistik, Untersuchung des Verteilungsmusters, bivariate und partielle Korrelationen und nichtparametrische Tests, wie z. B.* U*-Test nach Mann und Whitney, bei nicht normalverteilten Variablen.

## Ergebnisse

Die deskriptiv statistischen Eigenschaften der Patientenkohorte (*n* = 164) sind in Tab. 2 dargestellt. Bei der Frage nach den sportlichen Aktivitäten in der Vergangenheit gaben 82,3 % der Patienten (*n* = 135) an, zumindest selten Sport betrieben zu haben. Dabei haben 34 % (*n* = 56) darüber hinaus über ihre Teilnahme am Leistungs- und Vereinssport in den früheren Jahren berichtet. Bezüglich der früher betriebenen Sportart hat sich eine erhebliche Spannbreite gezeigt. Am häufigsten kamen in unserer Kohorte die Ballsportarten vor, diese haben etwa 18 % aller ehemaligen Sportler betrieben, gefolgt von Rückschlagsportarten (16 %), Schwimmen und Radsport (je 12,5 %). Weitere Sportarten waren schwächer repräsentiert. Die durchschnittliche wöchentliche Trainingszeit bei den ehemaligen Leistungssportlern betrug sportartübergreifend 6 h.

**Table 2:** 

Parameter		Wert
*n*		164
Alter, J, MW ± SD (min–max)		58,3 ± 13,7 (21–86)
Erkrankungsdauer, Mo, MW ± SD (min–max)		169 ± 127 (0–713)
Frauen, *n* (%)		127 (77)
BMI, kg/m^2^, MW ± SD (min–max)		26,4 ± 4,7 (17,3–43)
Ernährungszustand nach BMI, *n* (%)	Untergewichtig	5 (3)
	Normalgewichtig	74 (45,1)
	Übergewichtig	55 (33,5)
	Adipös	30 (18,3)
Ernährungsverhalten, *n* (%)	Ohne Restriktion	150 (91,5)
	Vegetarisch	13 (7,9)
	Vegan	1 (0,6)
Rauchen, *n* (%)	Nie geraucht	86 (52,4)
	Ex-Raucher	52 (31,7)
	Raucher	26 (15,9)
Alkoholkonsum, g pro Woche, *n* (%), MW ± SD (min–max)		67 (40,9), 27,1 ± 22,8 (0–84)
Kein Alkoholkonsum, *n* (%)		97 (59,1)
DAS28, MW ± SD (min–max)		2,9 ± 1,2 (0,3–7,8)
Krankheitsaktivität, *n* (%)	Niedrig (DAS28 < 3,2)	113 (68,9)
	Moderat (DAS28 3,2–5,1)	41 (25,0)
	Hoch (DAS28 > 5,1)	10 (6,1)
Körperliche Aktivität, *n* (%)	Niedrig	64 (39)
	Moderat	60 (36,6)
	Hoch	40 (24,4)
Medikamentöse Therapie, *n* (%)	*Synthetische DMARDs*	141 (86)
	Kinasehemmer	15 (9,1)
	*Biologika*	78 (47,6)
	*Analgetika*	77 (47)
	NSAR	53 (32,3)
	Opioide	15 (9,1)
Funktionstraining, *n* (%)		32 (19,5)

*J* Jahre, *Mo* Monate, *BMI* Body-Mass-Index, *kg* Kilogramm, *m*^*2*^ Quadratmeter, *MW* Mittelwert, *DAS28* Disease Activity Score 28, *DMARDs* „disease modifying anti-rheumatic drugs“, *NSAR* nichtsteroidale Antirheumatika, *SD* Standardabweichung, *n* Anzahl, min Minimum, *max* Maximum, *g* Gramm

Bei der Frage nach den aktuellen sportlichen Aktivitäten haben 57,9 % der Patienten (*n* = 95) angegeben, mindestens 30 min wöchentlich Sport zu treiben. Hier stellen die 3 häufigsten sportlichen Betätigungen die Übungen zur Stabilisierung der Körpermuskulatur in Form von Gymnastik (35 % der sportlich aktiven Personen), auch im Wasser bzw. Schwimmen (18 %) und Radfahren (13 %) dar. Außerdem wurden Nordic Walking (12 %), Fitnessstudioaktivitäten (8 %) und Wandern (7 %) angegeben.

Die antirheumatische Therapie wurde leitliniengerecht durchgeführt, einzelne Medikamentengruppen sind in der Tab. 2 aufgelistet. Der Anteil der Patienten, die sedierende Analgetika erhielten, betrug 9 % (*n* = 15); 32 Patienten (19,5 %) erhielten Funktionstraining. Ein signifikanter Zusammenhang zwischen der Einnahme von sedierenden Medikamenten und der körperlichen Aktivität konnte nicht gesehen werden (*p* = 0,697).

## Körperliche Aktivität, Funktionskapazität und Fatigue

Nach körperlicher Aktivität hat sich folgende Verteilung ergeben: 39 % (*n* = 64) der Patienten wiesen eine niedrige, 37 % (*n* = 60) eine moderate und 24 % (*n* = 40) eine hohe körperliche Aktivität auf. Der Unterschied zwischen der durchschnittlichen wöchentlichen MET-Minutenzahl bei Patienten mit niedriger und der mit hoher körperlicher Aktivität betrug 6163 MET-Minuten. Ein von EULAR als minimal empfohlenes wöchentliches MET-Minuten-Volumen von 500 [21] erreichten 64 % der Patienten (*n* = 105). Die Empfehlung einer moderaten körperlichen Aktivität von 30 min an 5 Tagen pro Woche bzw. einer hohen körperlichen Aktivität von 20 min an 3 Tagen pro Woche erfüllten 23 % der Patienten (*n* = 38). Von den 114 Patienten, die eine Frage zur täglichen Sitzdauer beantworteten, haben 28 % (*n* = 32) länger als 6 h täglich im Sitzen verbracht. Im untersuchten Kollektiv hing die tägliche durchschnittlich im Sitzen verbrachte Zeit nicht von dem Grad der körperlichen Aktivität ab. Bei Patienten mit hoher körperlicher Aktivität war diese Zeit tendenziell etwas länger als bei den Patienten mit niedriger körperlicher Aktivität (5,3 h vs. 4,2 h, *p* = 0,128, s. Tab. 3).

**Table 3:** 

Parameter		Körperliche Aktivität			Gesamt
		Niedrige	Moderate	Hohe	
*n*		64	60	40	164
Alter (J)	MW	63,9	54,4	55,1	58,3
	SD	13,1	12,5	13,8	13,7
Erkrankungsdauer (Mo)	MW	185,5	157,7	160,9	169,3
	SD	129,6	131,9	114,1	126,8
MET (Min/Wo)	MW	275,3	1913,3	6437,9	2377,7
	SD	447,8	1182,1	3864,7	3166,7
Zeit im Sitzen (h)^a^	MW	4,2	5,2	5,3	4,9
	SD	3,3	2,7	2,9	3,0
FFbH-Wert (%)	MW	58,3	75,4	83,4	70,7
	SD	26,1	23,1	17,5	25,3
GFI-Wert	MW	25,7	24,0	16,6	22,9
	SD	12,0	10,1	10,5	11,5
DAS28-Wert	MW	3,1	2,8	2,6	2,9
	SD	1,4	1,0	1,3	1,2
CRP (mg/l)	MW	6,1	4,7	5,2	5,4
	SD	8,7	7,3	9,8	8,4
BKS (mm/h)	MW	23,0	14,4	15,7	18,1
	SD	23,8	10,0	13,7	17,8
VAS (mm)	MW	49,2	48,7	38,3	46,3
	SD	21,1	21	20,6	21,3
BMI-Wert (kg/m^2^)	MW	27,3	26,6	24,4	26,4
	SD	5,7	4,2	3,1	4,7

*n* Anzahl, *MET* metabolisches Äquivalent, *Min* Minuten, *Wo* Woche, *h* Stunde, *MW* Mittelwert, *SD* Standardabweichung, *J* Jahre, *Mo* Monate, *FFbH* Funktionsfragebogen Hannover, *GFI* Global Fatigue Index, *DAS28* Disease Activity Score 28, *CRP* C-reaktives Protein, *BKS* Blutkörperchensenkungsgeschwindigkeit, *VAS* Visual Analog Scale, *BMI* Body-Mass-Index, *kg* Kilogramm, *m*^*2*^ Quadratmeter, *mm* Millimeter, *mg/l* Milligramm pro Liter, *mm/h* Millimeter pro Stunde

^a^*n* = 114

Patienten mit hoher und moderater körperlicher Aktivität waren durchschnittlich 9 Jahre jünger als Patienten mit niedriger körperlicher Aktivität (*p* < 0,001). Der durchschnittliche Unterschied in der Erkrankungsdauer zwischen den Gruppen mit der hohen und der niedrigen körperlichen Aktivität betrug ca. 2 Jahre und war nicht signifikant (*p* = 0,327).

Der durchschnittliche BMI lag im Gesamtkollektiv bei 26,4. Normalgewichtig (BMI 20–24,9) waren 45,1 % der Patienten (*n* = 74). Der durchschnittliche BMI-Wert bei Patienten mit niedriger körperlicher Aktivität war mit 27,3 ca. 12 % höher als bei den Patienten mit hoher körperlicher Aktivität (24,4). Die körperliche Aktivität korrelierte im untersuchten Patientenkollektiv signifikant sowohl mit dem Alter (*p* < 0,001) als auch mit dem BMI (*p* = 0,018).

Der Anteil der Patienten mit relevanter Funktionsbeeinträchtigung (Funktionskapazität <60 %) lag bei 30,5 % (*n* = 50), gleichzeitig wiesen 46,3 % der Patienten (*n* = 76) eine normale Funktionskapazität (>80 %) auf. Knapp ein Viertel der Patienten (23,2 %, *n* = 38) zeigte eine leichte bis mäßige Funktionsbeeinträchtigung. Die steigende körperliche Aktivität entsprach einer Zunahme der FFbH-Werte. Bei Patienten mit hoher körperlicher Aktivität lag der durchschnittliche FFbH-Wert bei 83,4 %, entsprechend einer unbeeinträchtigten Funktionskapazität. Patienten mit niedriger körperlicher Aktivität zeigten dagegen eine relevante Funktionsbeeinträchtigung (FFbH-Mittelwert 58,3 %).

Der Mittelwert vom GFI betrug im untersuchten Patientenkollektiv 22,9 (1–49,8). Der GFI-Wert von 20 wurde von 56,7 % der Patienten (*n* = 93) überschritten. Patienten mit hoher körperlicher Aktivität wiesen die niedrigsten GFI-Werte auf (Mittelwert 16,6). Bei den Patienten mit niedriger körperlicher Aktivität war der mittlere GFI-Wert um 55 % höher (25,7).

Somit korreliert die körperliche Aktivität einerseits mit der Funktionskapazität und andererseits mit dem GFI (jeweils *p* < 0,001). Die Signifikanz der Assoziation bleibt auch nach Berücksichtigung des Alters und der Erkrankungsdauer als Störvariablen bestehen.

Der durchschnittliche DAS28-Wert im Gesamtkollektiv betrug 2,9 ± 1,2. Der Wert von 2,9 entspricht einer niedrigen Krankheitsaktivität. Patienten mit hoher körperlicher Aktivität haben einen durchschnittlichen DAS28-Wert von 2,6. Nach EULAR-Kriterien entspricht dieser Wert einer Remission. Die Abnahme der körperlichen Aktivität korrelierte mit einer steigenden Krankheitsaktivität (*p* = 0,045). Unter Berücksichtigung des Alters war diese Tendenz allerdings nicht statistisch relevant (*p* = 0,119). Der DAS28-Mittelwert liegt bei Patienten mit niedriger körperlicher Aktivität bei 3,1 (um 20 % höher). Umgekehrt wiesen 60 % der Patienten (*n* = 6) mit hoher Krankheitsaktivität (DAS28 > 5,1) aus unserer Kohorte eine niedrige körperliche Aktivität auf. Weder vom Alter noch von der Erkrankungsdauer beeinflusst, fiel die Einschätzung des eigenen Krankheitszustandes, angegeben in Form von visueller analoger Skala (VAS), signifikant unterschiedlich bei Patienten mit niedriger und mit hoher körperlicher Aktivität aus (*p* = 0,008).

Laborparameter wie Blutkörperchensenkungsgeschwindigkeit (BKS) und C‑reaktives Protein (CRP) zeigten keinen Zusammenhang mit der körperlichen Aktivität (*p* = 0,187 und *p* = 0,158). Der Leistungssport in der Vergangenheit war nicht mit der aktuellen sportlichen Aktivität assoziiert (*p* = 0,130). Die Art der Ernährung und der Therapie, der Tabak- und Alkoholkonsum zeigten ebenfalls keinen Zusammenhang mit dem Niveau der körperlichen Aktivität.

## Diskussion

In unserer Querschnittanalyse konnte gezeigt werden, dass die aktuelle körperliche Aktivität der Patienten einerseits hoch signifikant mit der Funktionskapazität und andererseits mit dem GFI korreliert (*p* < 0,001). Weiterhin ließen sich signifikante Assoziationen mit der Krankheitsaktivität (*p* = 0,045) und dem BMI (*p* = 0,018) feststellen. Unsere Ergebnisse können teilweise frühere Daten stützen, die zeigten, dass eine regelmäßige sportliche Betätigung, speziell bei den Patienten mit rheumatoider Arthritis, eine Reihe spezifischer Vorteile mit sich bringen kann. Auch wenn die untersuchten Gruppen sehr klein waren, so konnte dennoch gezeigt werden, dass sportliche Aktivität nicht nur zur Reduktion der Krankheitsaktivität und der Fatigue beitragen kann, sondern auch Schmerz lindern, Funktionskapazität verbessern und sich positiv auf den mentalen Zustand der Patienten auswirken kann [12]. Es muss an dieser Stelle allerdings auch betont werden, dass es nach wie vor wenig Informationen darüber gibt, wie viel körperliche Aktivität für Patienten mit rheumatoider Arthritis angemessen ist [30], und die Datenlage, inwiefern Sport Einfluss auf Müdigkeit, Krankheitsaktivität und Schmerz haben kann, nach wie vor sehr dünn ist.

Die Patienten aus unserem untersuchten Kollektiv mit hoher körperlicher Aktivität wiesen eine signifikant niedrigere Krankheitsaktivität auf. In der Literatur wird betont, dass der Grad der körperlichen Aktivität als guter Marker der Krankheitsaktivität bei rheumatoider Arthritis gelten kann [27, 29]. Andererseits gibt es Hinweise, dass das Niveau der körperlichen Aktivität in der Zeit vor der aktiven Erkrankung die Erkrankungsschwere beeinflussen kann [24]. Ergebnisse aus unserer Untersuchung zeigten diesbezüglich allerdings keine Korrelation zwischen der aktuellen Krankheitsaktivität und dem Leistungssport in der Vergangenheit bei den untersuchten Patienten. Der fehlende Zusammenhang kann hier evtl. dadurch erklärt werden, dass der Abstand zwischen der aktiven Sportzeit und dem Ausbruch der Erkrankung in unserer Untersuchung nicht definiert war. Dennoch gibt es Hinweise, dass die körperliche Aktivität die Entwicklung der rheumatoiden Arthritis verzögern kann [6].

In unserer Untersuchung an RA-Patienten war Leistungssport in der Vergangenheit nicht mit der aktuellen sportlichen Aktivität assoziiert. Für Personen ohne rheumatologische Grunderkrankung wurde dagegen eine Assoziation zwischen dem Leistungssport in den jungen Jahren und sportlicher Aktivität im mittleren Erwachsenenalter beschrieben [2]. Gegebenenfalls hat die Diagnosestellung einen entscheidenden Einfluss auf die sportliche Motivation der Patienten. Patienten mit rheumatoider Arthritis neigen dazu, infolge von Schmerzen und Fatigue weniger aktiv zu sein [10]. Insbesondere kann der Schmerz dazu führen, dass die Patienten Bewegung meiden und eine sog. Kinesiophobie entwickeln [11].

Auch Fatigue wird von der Mehrheit der RA-Patienten als ein relevantes Problem gemeldet [14]. In unserer Kohorte gaben 93 Patienten (56,7 %) Fatigue an. Die Patienten mit der ausgeprägtesten Fatigue waren auch am wenigsten aktiv. Fatigue hat einen bedeutenden Einfluss auf den allgemeinen Gesundheitszustand, die körperliche Aktivität und soziale Kommunikation der Patienten. Im Umkehrschluss hatten Patienten mit hoher körperlicher Aktivität aus unserem Kollektiv signifikant niedrigere GFI-Werte. Die Beobachtung deckt sich mit den Daten aus der aktuellen Literatur [12, 31]. Letztendlich wird auch von einem positiven Einfluss der körperlichen Aktivität auf die Depression berichtet [16]. Das subjektive Krankheitsempfinden korrelierte in unseren Untersuchungen unabhängig von Alter, Erkrankungsdauer und DAS28 ebenfalls mit der körperlichen Aktivität (*p* = 0,008), sodass im Umkehrschluss Gesundheitsedukation, Motivationstraining und eine gesunde psychische Verfassung Einfluss darauf haben könnten, die Schwelle zur physischen Aktivität zu senken. Sedierende Medikamente zeigten hingegen keinen signifikanten Einfluss auf die körperliche Aktivität (*p* = 0,697).

In unserem Patientenkollektiv wiesen körperlich aktive Personen eine uneingeschränkte Funktionskapazität auf, der durchschnittliche Prozentsatz des FFbH-Wertes betrug bei den Patienten mit hoher körperlicher Aktivität 83,4 %. Gleichzeitig zeigte sich dieser Wert bei Patienten mit niedriger körperlicher Aktivität mit 58,3 %, entsprechend einer relevanten Funktionsbeeinträchtigung. Dies weist einmal mehr darauf hin, wie wichtig es ist, Funktionseinschränkungen zu verhindern.

In Bezug auf die im Sitzen verbrachte Zeit während des Tages zeigten sich keine Unterschiede zwischen den Patienten mit hoher und den mit niedriger körperlicher Aktivität. Diese Beobachtung deckt sich mit den Daten aus aktueller Literatur [19]. Dennoch zeigt dauerhaftes Sitzen selbst unter körperlich aktiven Menschen eine strenge Assoziation mit erhöhtem Mortalitätsrisiko [15]. Deshalb werden nicht nur Bemühungen zur Steigerung der körperlichen Aktivität, sondern auch unabhängig davon explizite Anstrengungen zur Reduktion der im Sitzen verbrachten Zeit von Bedeutung sein. Hier könnten z. B. Bewegungspausen am Arbeitsplatz ein Modell sein, welches nachhaltig eine Verbesserung der körperlichen Aktivität und somit Gesamtmorbidität bewirken könnte.

Die aktuellen EULAR Empfehlungen [21] sind an den Empfehlungen zur sportlichen Aktivität der Weltgesundheitsorganisation (WHO), des American College of Sports Medicine (ACSM) und der American Heart Association (AHA) für gesunde Erwachsene angelehnt [7, 9, 32]. Demnach sollten Patienten mit rheumatologischen Erkrankungen ein wöchentliches MET-Minuten-Volumen von mindestens 500 erreichen, aus unserer Kohorte trifft dies für 64 % der Patienten (*n* = 105) zu. Die Empfehlung einer moderaten körperlichen Aktivität von 30 min an 5 Tagen pro Woche bzw. einer hohen körperlichen Aktivität von 20 min an 3 Tagen pro Woche erfüllten 23 % unserer Patienten (*n* = 38). Die Ergebnisse zeigen, dass zwar schon ein großer Teil den Empfehlungen nachkommt, aber durchaus noch Potenzial zur Verbesserung vorhanden ist. In der Allgemeinbevölkerung kommen allerdings auch nur 26 % der Männer und 19 % der Frauen den aktuellen Empfehlungen nach [23]. Unsere Daten belegen einen Zusammenhang zwischen höherem BMI und Alter bezüglich reduzierter körperlicher Aktivität. In einem 2019 veröffentlichen Review-Artikel wurde gezeigt, dass diese Assoziation in den meisten diesbezüglich untersuchten Kohorten zutrifft [3].

In der Vergangenheit wurde Patienten mit rheumatoider Arthritis häufig empfohlen, körperliche Aktivität zu vermeiden bzw. zu begrenzen aus Angst, die Krankheitssymptome und Gelenkdestruktionen zu verschlimmern [26]. Inzwischen hat das Wissen über die positiven Auswirkungen der körperlichen Aktivität den Einzug in sämtliche Richtlinien für die Betreuung der Patienten mit rheumatoider Arthritis gefunden: „In Anbetracht der klaren Evidenz für ihre Effektivität, Durchführbarkeit und Sicherheit werden Maßnahmen zur körperlichen Aktivität als integraler Bestandteil der Patientenversorgung erachtet.“ [21] Dennoch nutzen die Patienten mit rheumatoider Arthritis die physiotherapeutischen Angebote für die Erhöhung ihrer körperlichen Aktivität nicht im ausreichenden Maß [18, 26]. RA-Patienten fehlt oft immer noch das ausreichende Bewusstsein über die positiven Auswirkungen der körperlichen Aktivität, hinzu kommt eine fehlende Motivation. Andererseits schränken auch Aufklärungsdefizite beim medizinischen Personal und unzureichende Zugänglichkeit der Bewegungsangebote deren Verbreitung unter den RA-Patienten ein [30]. Oft müssen Patienten sehr lange auf freie Plätze in Krankenkassen-unterstützten Trainingskursen warten. Hier wäre der Ausbau der geförderten Modelle wünschenswert. Andererseits könnten Patienten dazu motiviert werden, in eigener Initiative Gymnastik oder leichten Ausdauersport zu betreiben, auch wenn es kein fremdfinanziertes Angebot gibt. In unserer eigenen Kohorte gaben nur 19,5 % der Patienten an, an Funktionstraining oder Rehasport-Maßnahmen teilzunehmen, sodass wir in unserer Institution diesbezüglich eine Verbesserung der Verschreibung anstreben.

Bemühungen zur Überwindung dieser Hürden sollten angestrebt werden, um den Anteil der Patienten mit niedriger körperlicher Aktivität zu senken. Auf der einen Seite können der gezielte physiotherapeutische Einsatz, das Funktionstraining, der Ausbau der physiotherapeutischen oder vereinssportlichen Bewegungsangebote die Schwelle für den rechtzeitigen Beginn der Aktivitäten senken. Gleichzeitig soll durch Aufklärung der Patienten das Bewusstsein bezüglich der Wichtigkeit der körperlichen Aktivität gesteigert werden. Auf der anderen Seite wären gezielte Fort- und Weiterbildungen in Bezug auf den therapeutischen Nutzen der körperlichen Aktivität beim ärztlichen und pflegerischen Personal erstrebenswert.

Unsere Querschnittstudie ist durch mehrere Faktoren in ihrer Aussagekraft limitiert, und bei der Interpretation der Daten ist aufgrund der multiplen Einflussgrößen auf die körperliche Aktivität und der verhältnismäßig kleinen Fallzahl Vorsicht geboten. Da es sich um keine Interventionsstudie, sondern um eine Querschnittanalyse handelte, lässt sich die Frage nach Kausalität des Zusammenhanges nicht beantworten. Letztendlich konnte unsere Studie zeigen, dass die körperliche Aktivität mit sehr vielen Faktoren, z. B. BMI, Alter, Funktionskapazität, Fatigue und auch mit subjektivem Krankheitsempfinden korreliert. Ein positiver Einfluss auf die Krankheitsaktivität und psychische Verfassung scheint im Umkehrschluss wahrscheinlich, kann durch unsere Daten jedoch nur spekulativ vermutet und nicht belegt werden. Größere Longitudinalstudien sind zur Beantwortung dieser Fragestellungen nötig.

Unsere Ergebnisse betonen dennoch die Notwendigkeit, die aktuellen EULAR-Empfehlungen zur Bewegung umzusetzen, und können als Aufforderung und Motivation zur sportlichen Aktivität unserer Patienten verstanden werden. Gleichzeitig richtet sich unser Appell auch an alle betreuenden Ärzte von Patienten mit rheumatologischen Erkrankungen, Patienten nicht nur zu motivieren, sondern ihnen auch Wege zur sportlichen Aktivität aufzuzeigen und sie zur Teilnahme an Sportprogrammen aktiv zu unterstützen.

## Fazit für die Praxis

- Körperliche Aktivität ist mit höherer Funktionskapazität und weniger Fatigue bei Patienten mit rheumatoider Arthritis assoziiert.
- Patienten mit rheumatologischen Erkrankungen sollten zu sportlichen Aktivitäten motiviert werden.
- Die Möglichkeiten des physiotherapeutischen Einsatzes und des Funktionstrainings sollten ausgeschöpft werden.

## Caption Electronic Supplementary Material




