# Supplementary material for: Association of physical activity with fatigue and functional capacity in patients with rheumatoid arthritis
Source: Z Rheumatol. 2020 Jun 25;80(2):113–21. [Article in German] doi: 10.1007/s00393-020-00830-2 (PMC8426241; doi:10.1007/s00393-020-00830-2)
Supplement: Supplementary file 1 [file 393_2020_830_MOESM1_ESM.pdf]

Elektronisches Zusatzmaterial

Auswertung des Fragebogens Multidimensional Assessment of Fatigue (MAF)

Der Bogen beinhaltet 16 Fragen. Fragen 1 und 4-14 werden auf einer Skala von 1 („ganz und gar nicht“) bis 10 („sehr stark“), Frage 2 von 1 („mild“) bis 10 („stark“), Frage 3 von 1 („keine Not“) bis 10 („maximale Not“) beantwortet. Fragen 15 und 16, die sich mit der Dynamik der Müdigkeit in der letzten Woche beschäftigen, werden auf einer Skala von 1 bis 4 beantwortet. Fragen 1 bis 15 sind für die Ermittlung des globalen Müdigkeits-Index geeignet. Für diese Kalkulation ist es erforderlich, dass die Antwort auf Frage 15 zunächst in eine 1-10-Kategorie konvertiert wird, hierfür wird sie mit 2,5 multipliziert. Der Wert wird dann mit den Punktwerten der Antworten 1, 2, 3 und dem Mittelwert der gültigen Antworten 4-14 addiert. Sollte die Frage 1 mit 1 („keine Müdigkeit“) beantwortet werden, wird den Antworten 2-16 eine 0 zugewiesen. Die Kalkulation ergibt einen Punktwert im Bereich von 1 (keine Müdigkeit) bis 50 (maximale Müdigkeit).

Quelle: Belza, B. (1995). "Comparison of self-reported fatigue in rheumatoid arthritis and controls." The Journal of Rheumatology 22(4): 639-643.

Auswertung des Funktionsfragebogens Hannover (FFbH)

Der Bogen umfasst 18 Fragen. Jede Frage beginnt mit „Können Sie...?“. Als Antwortmöglichkeit gibt es einheitlich eine Skala mit den Alternativen „Ja“ (2 Punkte), „Ja, aber mit Mühe“ (1 Punkt) und „Nein oder nur mit fremder Hilfe“ (0 Punkte). Die Antworten der Einzelfragen werden aufsummiert, der Wertebereich liegt somit bei FFbH zwischen 0 und 36 Punkten. Im Ergebnis wird die Funktionskapazität als Gesamtwert von 0% (minimale Funktionskapazität) bis 100% (maximale Funktionskapazität) ausgegeben.

$$Funktionskapazität\ (\%) = \frac{Summe\ Punkte\ \times 2}{2 \times Anzahl\ der\ gültigen\ Antworten}$$

Quelle: Bengel, J., et al. (2008). Diagnostische Verfahren in der Rehabilitation, Hogrefe Verlag.
